# Preparation and Performance of Cement Mortar Reinforced by Modified Bamboo Fibers

**DOI:** 10.3390/polym12112650

**Published:** 2020-11-11

**Authors:** Yang Ban, Wei Zhi, Mingen Fei, Wendi Liu, Demei Yu, Tengfei Fu, Renhui Qiu

**Affiliations:** 1 College of Transportation and Civil Engineering, Fujian Agriculture and Forestry University, Fuzhou 350108, China; bonnybanban@163.com (Y.B.); zhiwei20200928@gmail.com (W.Z.); fmesaito@163.com (M.F.); wendi.liu@fafu.edu.cn (W.L.); yudemei0826@fafu.edu.cn (D.Y.); 2 CSCEC Strait Construction and Development CO., Ltd., Fuzhou 350015, China

**Keywords:** bamboo fiber, surface treatment, cement mortar, mechanical properties, durability, interfacial bonding

## Abstract

This study aims to prepare bamboo-fiber-reinforced cement composites and provide a solution to the issue of poor interfacial adhesion between bamboo fibers and cement matrix. The original bamboo fibers were modified by three moderately low-cost and easy-to-handle treatments including glycerol, aluminate ester, and silane treatments. The performance of the modified bamboo-fiber-reinforced cement composites was evaluated by a series of mechanical and durability tests, including flexural and compressive strength, water absorption, chloride ion penetration, drying shrinkage, freeze–thaw resistance, and carbonization. In addition, the microstructures of composites were characterized using a scanning electron microscope (SEM). The results showed that the composites reinforced with glycerol-modified bamboo fibers had 14% increased flexural strength and comparable compressive strength. From durability perspectives, all treatments showed similar performance in drying shrinkage, whereas aluminate ester treatment was the most effective in terms of impermeability, chloride resistance, freeze–thaw resistance, and carbonization. The results could provide insights to efficient and effective natural fiber treatment to enable better performance of natural-fiber-reinforced cement-based materials.

## 1. Introduction

Fiber reinforcement of cement-based materials has been a practice for many decades [1,2]. Many different types of fiber, such as metallic fiber, glass fiber, carbon fibers, synthetic polymer fiber, and natural fiber, have been proved beneficial [2]. In general, fibers could overcome shortcomings of cement-based materials such as low tensile strength, low toughness, and cracking issues, which is due to the crack-bridging mechanism where fibers can delay or prevent crack propagation in the fiber–cement composites [3,4]. In consideration of energy and environmental issues, renewable, biodegradable, and abundant bamboo fibers have been drawing more and more attention for their use as the reinforcement for cement-based composites.

Bamboo fiber is composed of multiple layers of axially distributed microfibrils which yield high tensile strength (639–813 MPa) with low density (1.38 g/cm^3^) [5]. The aspect ratio of single bamboo fiber is significantly higher than that of most wood fibers, and its specific strength and specific modulus are higher than those of glass fiber [5]. Therefore, bamboo fibers have the potential to be used in cement-based materials as a promising alternative reinforcement to synthetic polymer fiber and steel fiber. Since the pioneering work done by Pakotiprapha et al. [6] and Ramaswaymy et al. [7] in the 1980s, there have been a number of successful applications of bamboo fibers in cement-based materials [8,9,10,11,12,13,14,15]. In general, by adding bamboo fibers (or treated bamboo fibers), the reinforced cement composites could demonstrate comparable compressive strength, improved flexural or tensile strength, and usually much higher fracture toughness.

However, as a natural fiber, bamboo fiber needs to overcome the durability issue to be more effectively used in cement-based materials. The durability issue is related to high porosity of cement–fiber interface [16], which is caused by high water absorption of bamboo fiber and its dimensional instability due to humidity variation. The expansion and contraction of the fiber can weaken the bond between the fiber and the cement matrix [17,18,19]. However, the high alkalinity (pH > 13) in cement can be detrimental to bamboo fibers. Several studies have reported fiber degradation due to high alkalinity [20,21,22,23]. Under wetting/drying cycles, Mohr et al. [20] found fiber embrittlement due to portlandite precipitation in fiber lumen and/or fiber cell walls. Ardanuy et al. [21] also observed cement hydration compound deposits in the lumen and surface of cellulosic fibers.

Preventing the decline of fiber performance in high alkalinity is the key to a durable natural fiber cement-based composite. Fiber surface treatment is one necessary and effective method to increase physical and chemical stability [24,25]. Some treatments boil fibers in water [26], alkaline substances, [9,12,14,27], or other chemicals [28,29] to reduce the amount of water-soluble substances in the fiber. Other treatments modify the fiber surface with sodium carbonate [30], silane coating [31], and polymer modification [10,15,32] to enhance mechanical properties and durability of natural fibers in cement materials.

This study aims to prepare bamboo-fiber-reinforced cement mortar and provide potential solutions for durability issues due to poor interfacial adhesion between fibers and cement. The bamboo fibers used in this study were modified using surface treatments which are moderately low-cost and easy to handle. The microstructures of the composites were characterized using scanning electron microscopy (SEM). The performances of the modified bamboo-fiber-reinforced cement composites were evaluated in flexural and compressive strength, capillary water absorption (sorptivity), chloride penetration, drying shrinkage, freeze–thaw resistance, and carbonization.

## 2. Materials and Methods

In this study, bamboo fibers (either treated and untreated) were added to cement mortar. Materials used are introduced in the following sections.

### 2.1. Bamboo Fiber and Modification

**Figure 1:**
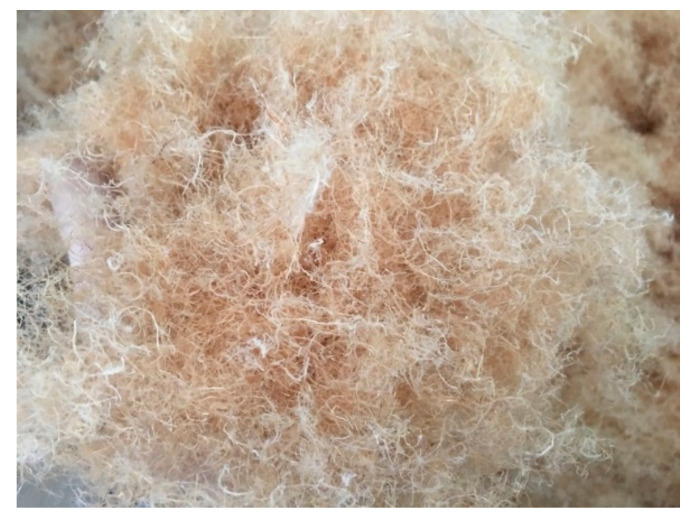
As-received raw bamboo fibers.

The raw bamboo fiber (as shown in Figure 1) was commercially available from a local manufacturer (HBS Chemical Technology, Nanping, Fujian) using locally harvested green bamboo (*Dendrocalamopsis oldhami* Munro). A proprietary method combining mechanical processing and biobased chemical solvent was used in the fiber manufacturing. The properties of the raw bamboo fiber are listed in Table 1.

**Figure 2:**
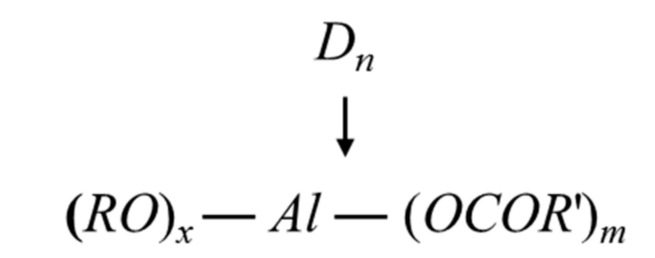
Chemical structure of aluminate ester (D_n_: dibutyl phthalate/dioctyl phthalate; RO: isopropyl group; OR’: isopropanol ester group).

Three modifiers were used in this study: a glycerol ethanol solution (1:1 by weight, Aladdin Chemicals, Shanghai), a liquid form (heated to 90 °C) aluminate ester solution (coupling agent, see Figure 2, Yuanke Chemicals, Fuzhou, Fujian), and a 98% KH-570 silane coupling agent (>98%, 3-methacryloxypropyltrimethoxysilane, Aladdin Chemicals, Shanghai, China), which were all purchased from Aladdin Chemicals (Shanghai, China). The reasons behind the selection of these modifiers were 1) they will potentially react with the hydroxyl groups on the surface of the bamboo fibers; 2) after applying the modifier, the surface should be partially hydrophobic and water-resistant in the meantime, which will theoretically increase the durability of the fiber without compromising the compatibility with the cement paste matrix; 3) the modifiers should be easily available and inexpensive.

Before applying the modifiers, raw bamboo fiber was dried in an oven at 110 °C for 6 h, and then opened and combed with a fiber carding machine. Approximately 900 g of combed fibers were then placed on wire mesh and 400 mL of modifier was evenly sprayed on the fiber surface using an electric spray gun (Power Action model SG9619, Ningbo, Zhejiang, China). To achieve uniform results, fibers should be tossed during the spray to make sure all surfaces were in contact with adequate modifiers. From many trials, a slightly dripping surface indicated a sufficient treatment. It should be noted that the aluminate ester solution was heated to 90 °C before applying to the fibers. Sprayed fiber was dried in the air for approximately 1 h and then moved to an oven at 110 °C for 12 h to ensure sufficient reactions at the surface. Dried fibers were then taken out and sealed in water-tight plastic bags for future use.

### 2.2. Preparation of Cement Mortar

In this study, a local P·O 42.5 ordinary Portland cement (Conch, Anhui, China) was used for cement mortar. The physical properties of the cement are listed in Table 2.

**Figure 3:**
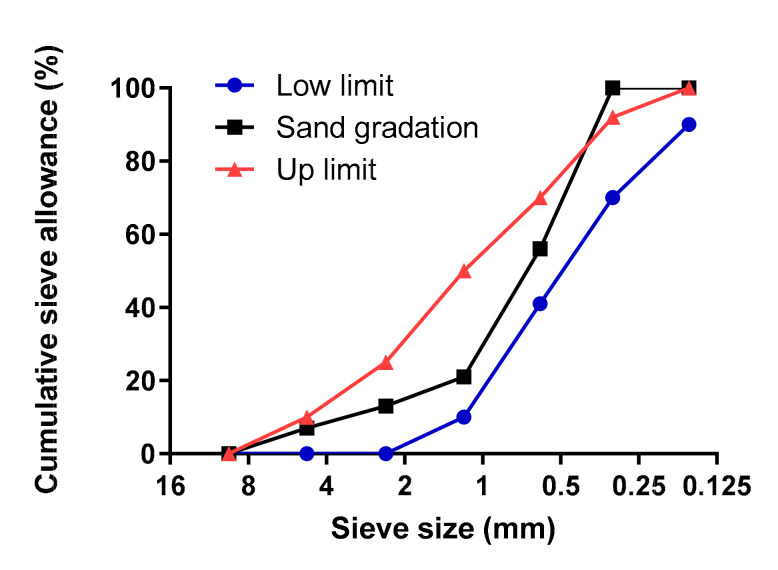
Sieve analysis of sand used in mortar.

The sieve analysis of sand used in mortar can be seen in Figure 3. Mixing was done in a mortar mixer. The mixture proportion is shown in Table 3. Water was added first to the bowl followed by cement. The mixer was set to low speed (140 ± 5 r/min) and the mixture was mixed for 60 s. Water reducer was added during the last 30 s. In the next 30 s, sand was slowly added to the bowl during mixing. Then, the mixer was stopped for about 15 s to scrape off paste on the blade and the side of the bowl. Then, the mixer was started again at low speed and the mixture was mixed for 90 s; fibers were slowly added during the mixing. After the mixing was stopped, mortar was poured into mold and consolidated on a vibration table for approximately 1 min. All samples were demolded at 24 h, then cured at 95% RH and 23 ± 2 °C until testing.

### 2.3. Experimental Methods

Flexural strength was tested on 40 mm × 40 mm × 160 mm third-point loading beams (ASTM C348 Standard Test Method for Flexural Strength of Hydraulic-Cement Mortar). After the flexural strength test, the broken pieces were used for the compressive strength test. For each mixture, at least 6 samples were tested. Scanning electron microscopy (SEM) images were taken at the fracture surfaces.

For each mixture, three 40 mm × 40 mm × 160 mm prisms were used for the drying shrinkage test (JGJ/T 70-2009 Standard for Test Method of Basic Properties of Construction Mortar). When an age of 28 d was reached, the samples were transferred to a curing box at 60 ± 5% RH and 23 ± 2 °C. Length change was measured by a comparator at 1 d, 3 d, 7 d, 14 d, 28 d, 45 d, 60 d, 75 d, and 90 d.

Cubic samples (70.7 mm × 70.7 mm × 70.7 mm) were used for the water absorption test (ASTM C1585 Measurement of Rate of Absorption of Water by Hydraulic Cement Concretes). At 28 d, cured samples were vacuum saturated, then placed in an environmental chamber at 80 ± 3% RH and 50 ± 2 °C for 72 h. Then, samples were sealed and stored at 23 ± 2 °C for another 15 days before the start of the absorption procedure. After conditioning, the side surfaces of the samples were sealed with paraffin wax, and the top surface was sealed with a loosely attached plastic sheet. Then, each sample was set on two glass rods in a container. Water was added to the container and the water level was maintained at approximately 3 mm above the bottom surface of the sample during the test. The mass change of the sample was monitored up to 3 days.

For chloride penetration test, same sample conditioning and test setup were used as the water absorption test by replacing water with 0.5% NaCl solution. After seven days, samples were transferred to a 50 °C oven and dried for 7 days. Powder samples were taken every 1.5 mm from the bottom surface to 9 mm height, then every 2.5 mm from 9 mm to 19 mm height. The obtained powder was grounded and passed through No. 30 sieve (0.6 mm mesh). Chloride ion content was determined by silver nitrate titration method (GB/T 50082-2009 Standard for Test Methods of Long-Term Performance and Durability of Ordinary Concrete).

The freeze–thaw test was carried out on 70.7 mm cubic samples (GB/T 50082-2009 Standard for Test Methods of Long-Term Performance and Durability of Ordinary Concrete). Samples were cured for 24 d, then stored under water at room temperature for 4 days. In the freeze–thaw testing machine, samples were slowly cooled to −18 °C in approximately 2 h, then stayed frozen for at least 4 h. During the thawing cycle, 20 °C water was added to the testing chamber to thaw the samples, and this thawing condition was kept for at least 4 h. At 25 cycles, samples at thawing condition were taken out for compressive strength test and mass loss test.

Carbonization test was done on 100 mm × 100 mm × 300 mm samples (GB/T 50082-2009 Standard for Test Methods of Long-Term Performance and Durability of Ordinary Concrete). Samples were taken out after 56 d of standard curing. Paraffin wax was used to seal all the surfaces except the two opposite longitudinal sides. Then, the samples were placed in an oven at 60 ± 2 °C to dry for 48 h. The dried specimen was placed in the carbonization chamber. Throughout the test, the concentration of carbon dioxide in the chamber was maintained at 20 ± 3%, the temperature was set at 20 ± 5 °C, and RH 70 ± 5%. Samples were taken out on 3 d, 7 d, 14 d, and 28 d. From one end of the prism, a 50 mm section was cut off and 1% phenolphthalein ethanol solution was sprayed on the surface after a proper cleansing. After 30 s, the carbonation depths of eight equidistant points on the sample was measured and the mean value was taken as the carbonation depth of the specimen at this age. The rest of the cut sample was put back to the carbonization chamber for further tests and the cut surface was also sealed with paraffin wax.

## 3. Results and Discussions

### 3.1. Mechanical Properties

**Figure 4:**
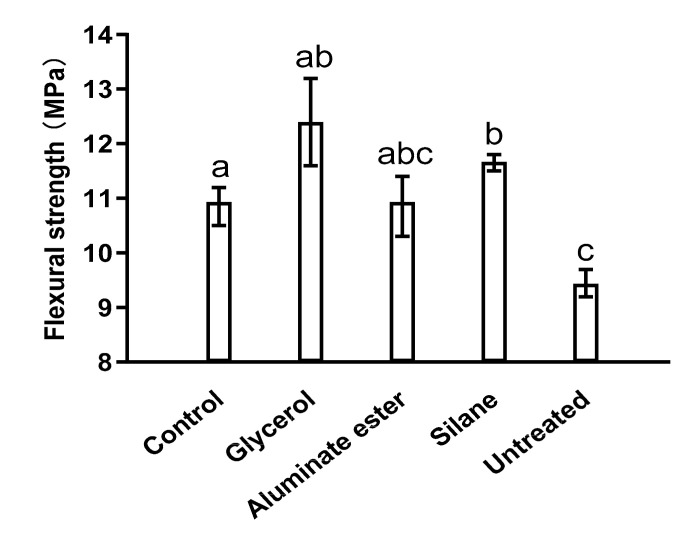
Flexural strength of different mixtures at 28 d. Note: Data were analyzed with one-way ANOVA. Significant differences exist between any two groups when they do not share a common letter over the columns. (This applies to the following figures.)

**Figure 5:**
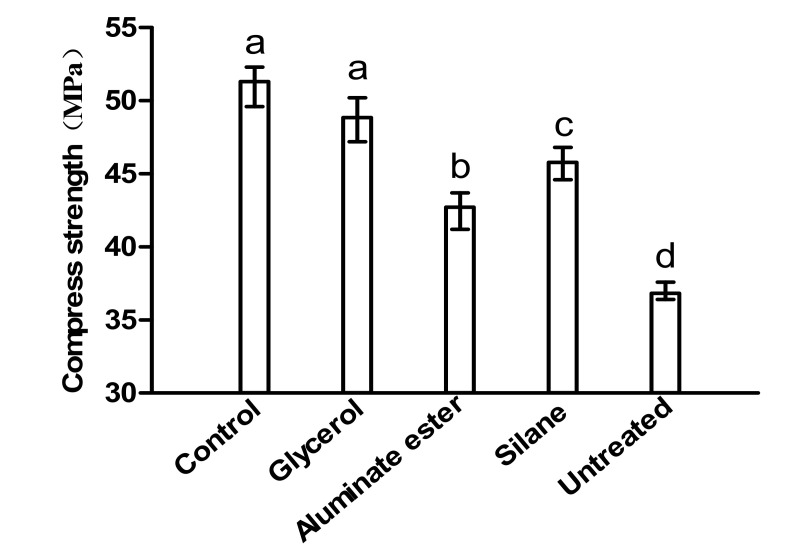
Compressive strength of different mixtures at 28 d.

The flexural strength and compressive strength results are shown in Figure 4 and Figure 5, respectively.

From both results, the strength of the untreated fiber is the lowest. There are two possible reasons. First, during the trial batches, it was observed that untreated fiber tended to agglomerate much more than treated fibers. Comparing to cement paste matrix and sand, bamboo fibers have large porosity and low rigidity. Agglomeration in the cement paste matrix would serve as defects and offset any benefits provided by the fiber reinforcement. In addition, surface treatment tended to toughen the bamboo fiber resulting in higher flexural strength. The glycerol mixture resulted in the most significant tensile strength increase (14%) on 28 days compared to the control mixture, followed by the silane mixture. However, the aluminate ester mixture did not show a significant increase in terms of tensile strength.

In terms of compressive strength, it should be pointed out that, other than the glycerol mixture, all other mixtures had significant resulting decreases in compressive strength. It is not unusual to observe that the addition of fibers could lower compressive strength. This is because the compressive strength of a given cementitious system is related to the total porosity. However, depending on applications, compressive strength of cementitious materials often has high redundancy, meaning an insignificant decrease in compressive strength is sometimes acceptable with the gain in other performance. The glycerol mixture demonstrated the best effectiveness both in tensile strength and compressive strength. It should also be noted that due to undesired mechanical properties of the untreated mixture, it was not included in the durability tests.

### 3.2. Durability Performance

#### 3.2.1. Drying Shrinkage

**Figure 6:**
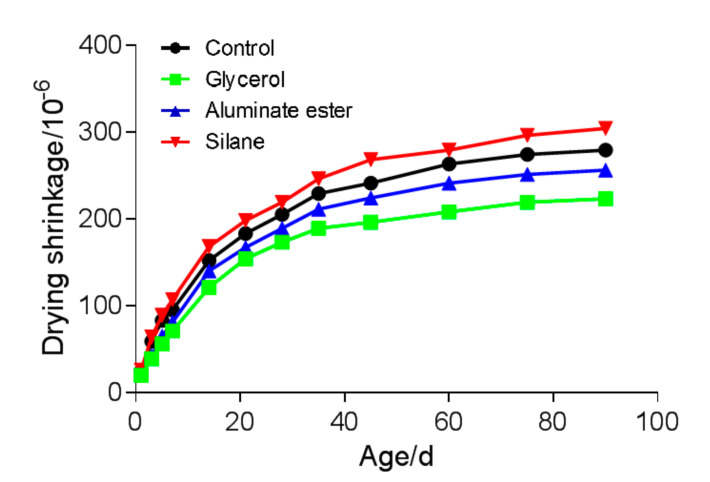
Drying shrinkage of different mixtures.

The drying shrinkages from four mixtures at different ages are shown in Figure 6, and a tabulated result can be seen in Table 4.

Unlike synthetic or metal fibers which are mostly solid, natural fibers such as bamboo fiber are porous. This is due to their fiber structures. One concern of using natural fibers in cement composite is that the connected pore structure could facilitate drying and, therefore, induce more drying shrinkage. The results showed that throughout all ages, the drying shrinkages, from the highest to the lowest, were those of the silane mixture, the control mixture, the aluminate ester mixture, and the glycerol mixture. Although the drying shrinkage of the silane mixture was slightly higher than that of the control mixture (9.0% at 90 d), it should be noted that all mixtures showed low shrinkage strain (< 350 μm/m). This result also provided indirect evidence that the modifying agent selected had high water resistance; therefore, water evaporation through the fiber structure was mostly prevented.

#### 3.2.2. Absorption Rate

**Figure 7:**
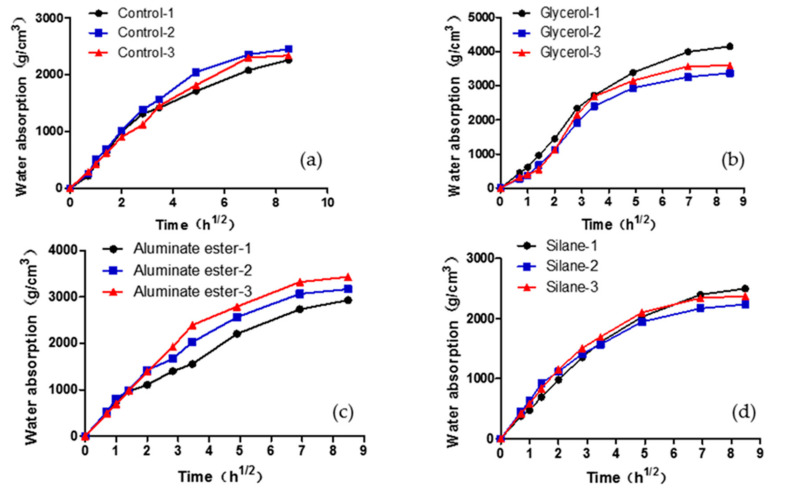
Capillary water absorption curve of (**a**) control mixture, (**b**) glycerol mixture, (**c**) aluminate -ester mixture, and (**d**) silane mixture.

The capillary water absorption coefficients of the four mixtures are listed in Table 5, which indicate water absorption rate per unit area. The coefficient was obtained by fitting the relationship between capillary water absorption and time (square root), as shown in Figure 7.

Different from drying shrinkage, the water absorption test measures sorptivity when the sample is in contact with external water. The results are often used to quantify concrete durability, which is a function of penetrability of the pore structures. In unsaturated samples (common condition of most concrete), the rate of ingress of water is controlled by sorptivity. A higher absorption rate usually indicates an open and connected pore system, which will facilitate water and other ions migrating into the inside of the concrete. A higher absorption rate also indicates that it takes shorter time for the concrete sample to reach critical saturation point at which freeze–thaw damage could occur.

The results showed that, other than the silane mixture, all other mixtures demonstrated similar initial water absorption coefficients. The silane mixture was 9% higher than the control mixture. This indicates that if impermeability is of critical concern for the concrete, silane might not be a proper modifying agenting for bamboo fiber. However, the reason why the silane mixture demonstrated higher sorptivity may be due to the hydrolysis reaction of excessive silane with water, and the excessive silane may have resulted from the residual of reaction with the hydroxyl groups of bamboo fibers [33].

#### 3.2.3. Chloride Penetration

**Figure 8:**
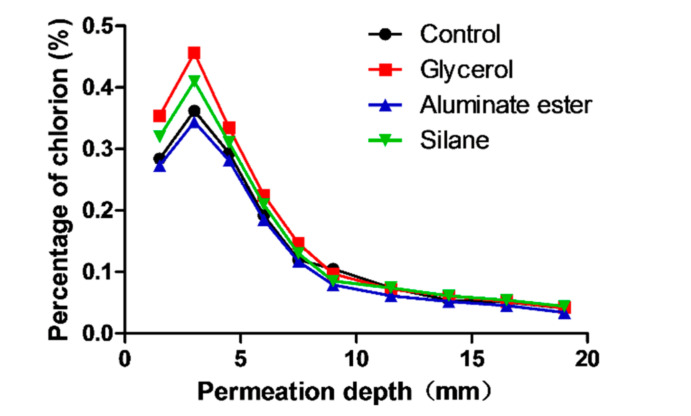
Chloride penetration depth due to sorptivity at 7 d.

The chloride penetration test used a similar setup as the absorption test, and showed similar results. The chloride concentrations of four mixtures at different depths are shown in Table 6 and Figure 8. It should be noted that in this test, chloride penetration was due to capillary action.

The content of chloride was higher near the surface with the presence of fiber, likely due to the connected pore structure provided by the fiber. With the increase of depth, the content of chloride ion decreased. When the penetration depth reached beyond 10 mm, the difference of chloride concentration became insignificant. However, at 19 mm, the silane mixture still showed a higher chloride concentration which aligned well with the absorption test results.

#### 3.2.4. Freeze–Thaw Resistance

The mass loss and compressive strength loss of four mixtures after 25 cycles of freezing and thawing (D25) are shown in Table 7. After the test, all samples were visually inspected and no noticeable spalling was observed. All samples met the freeze–thaw performance requirement (mass loss < 5%, and compressive strength loss < 25%). Compressive strength loss in the glycerol mixture (14.2%) and the silane mixture (12.2%) was much more significant than the control mixture (5.2%). On the contrary, the aluminate ester mixture demonstrated superior freeze–thaw resistance with 1.4% compressive strength loss. One possible reason is discussed in Section 3.3.

#### 3.2.5. Carbonization Test

**Figure 9:**
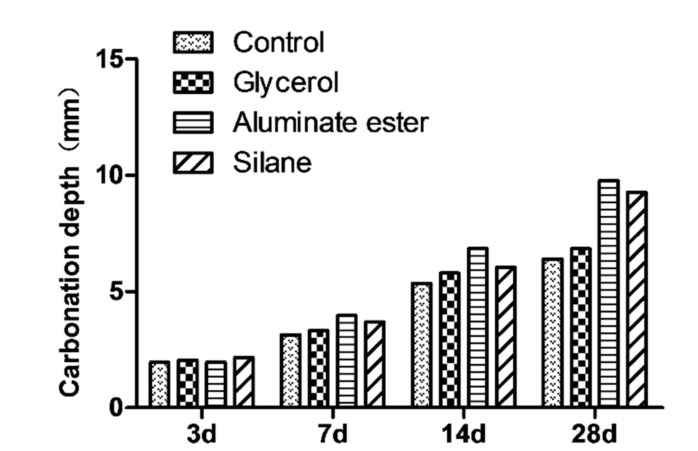
Carbonation depth of four mixtures at different age.

The carbonation depths of four mixtures at different ages are shown in Figure 9. At 28 d carbonization age, the glycerol mixture showed a carbonization depth comparable to that of the control mixture. This possibly indicated that glycerol-treated fiber had a denser interface between the fiber and the cement matrix, which aligns with the mechanical property results. However, the aluminate ester mixture and the silane mixture both demonstrated more than 50% more carbonized depth than the control mixture, which indicated that these two modifying agents might not be suitable for concrete application when carbonization is a critical concern.

### 3.3. Interfacial Bond

**Figure 10:**
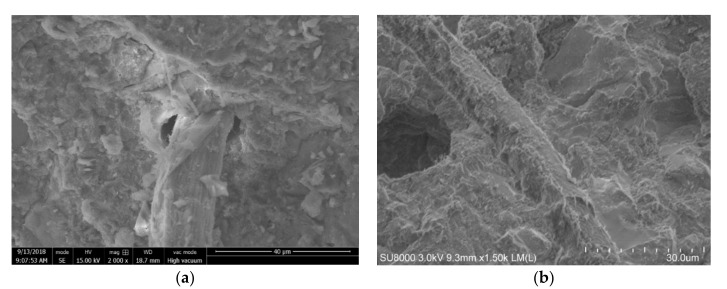

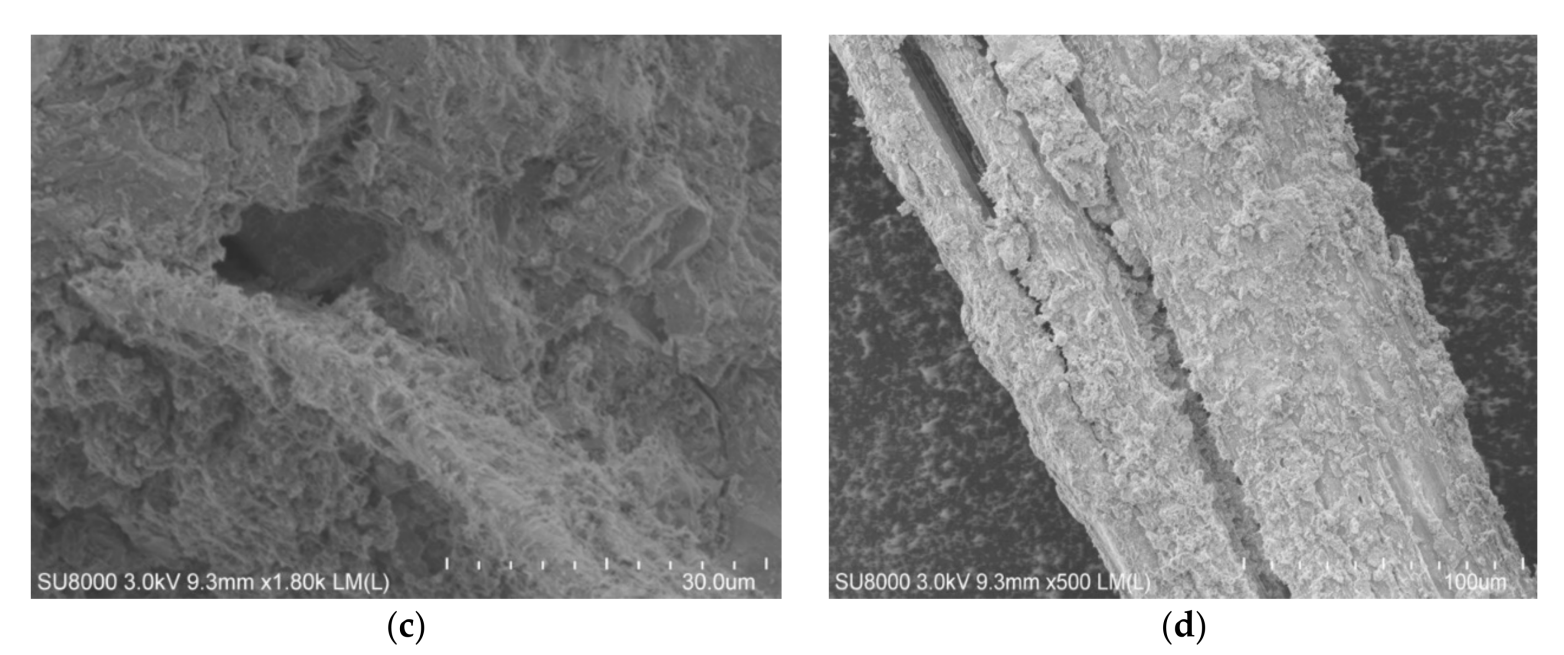
SEM images of the fractured surfaces of (**a**) untreated fiber, (**b**) fiber treated with aluminate ester, (**c**) fiber treated with glycerol, and (**d**) a single fiber treated with silane.

SEM images were taken from the fractured surface of tested flexural strength samples. Figure 10 shows a typical condition of fibers in the mortar. Figure 10a is the untreated fiber, which shows a debonding and a distinct interfacial boundary between the fiber surface and the cement matrix. Figure 10b,c shows the rough surface of aluminate ester treated fiber and glycerol treated fiber, and a visually good bonding between the fibers and the cement matrix was observed. This is a direct piece of evidence that that the modifying agent selected can improve the compatibility between the treated fiber surface and cement matrix. Figure 10d shows the surface of a single fiber treated by silane which indicates that the good bonding could likely be due to the comparatively rough surface from the treatment (compared to the untreated fiber).

**Figure 11:**
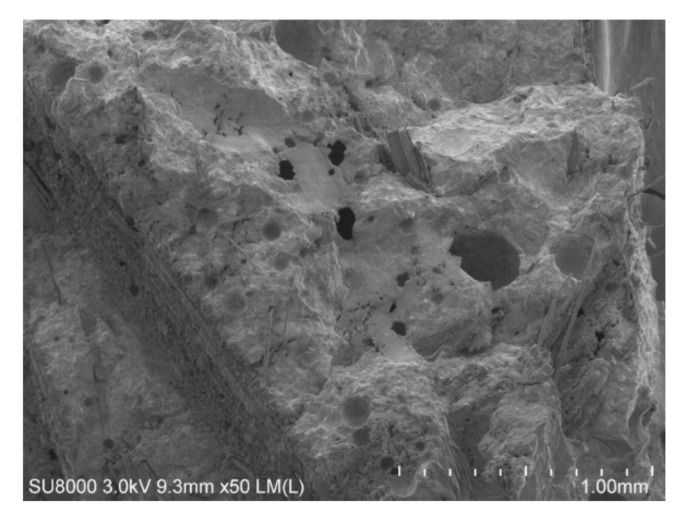
SEM images of fracture surface of fiber treated by aluminate ester.

Figure 11 shows an SEM image of the aluminate ester mixture. It is interesting to observe many small pores in the cement matrix. This could likely be due to the air entraining effect of aluminate ester during mixing. Therefore, the decrease in compressive strength can be explained by the fact that these small pores appear well distributed and connected, which could improve the freeze–thaw resistance of the sample. This observation aligns with the fact that the aluminate ester mixture demonstrated the best freeze–thaw resistance.

## 4. Conclusions

This study attempted to improve mechanical and durability performance of bamboo-fiber-reinforced cement mortar by modifying fibers using surface treatments (i.e., glycerol solution, aluminate ester, and silane) with moderately low cost and easy handling. The performances of the modified bamboo-fiber-reinforced cement composites were evaluated in terms of flexural and compressive strength, drying shrinkage, capillary water absorption, freeze–thaw resistance, chloride penetration, and carbonization. The following conclusions can be drawn:

1) In terms of mechanical properties, the glycerol-modified bamboo-fiber-reinforced cement composites demonstrated significantly higher flexural strength than the other mixtures, and compressive strength comparable to that of the control mixture. The glycerol mixture also showed the lowest drying shrinkage.
2) The results of water absorption and chloride ion erosion tests for composites showed that the addition of modified bamboo fibers influenced the impermeability greatly, and the aluminate-ester-modified bamboo fiber composites have the best impermeability among the three composites. The aluminate-ester-modified composites have a satisfactory freeze–thaw resistance, which is likely related to their air entrainment demonstrated by the SEM image. Adding aluminate-ester-modified bamboo fibers has the potential to effectively slow down the aging process and expand the service life of the composites.
3) The results of carbonization experiments showed that the aluminate ester mixture and the silane mixture might not be suitable for concrete application when carbonization is a critical concern.

Future work should include investigations of the mechanism of modifying agents on the fiber surface and the effect of the modifying agent on interfacial bonding. A more ideal modifying agent which is capable of improving both mechanical and durability performance is still needed to prepare high-performance bamboo-fiber-reinforced cement-based materials.

## Figures and Tables

**Table 1:** Properties of raw bamboo fiber.

Properties	Results
Chemical composition	Cellulose 65.6% Lignin 11.5% Pentosan 17.1%
Fiber elastic modulus (1% elongation, cN/dtex)	21.9
Fracture strength (cN/dtex)	2.44
Linear density (tex)	3.0
Density (g/cm^3^)	1.575
Breaking length (km)	8.6
Elongation at breaking (%)	2.58
Average length (cm)	3.0
Average diameter (μm)	28.03

**Table 2:** Physical properties of cement.

Properties		Limit	Test Results
Setting time	Initial	≥45 min	130 min
	Final	≤10 h	250 min
No. 200 Sieve (75 μm) residual		≤10.0	0.9
Flexural strength (MPa)	3d	≥3.5	4.7
	28d	≥6.5	8.4
Compressive strength (MPa)	3d	≥16	24.4
	28d	≥42.5	47.6
Density (g/cm^3^)			3.05

**Table 3:** Mixture proportions.

Mixture	Cement (kg/m^3^)	Sand (kg/m^3^)	Water (kg/m^3^)	Water Reducer (%)	Fiber (vol%)
Control	650	509	228	0.5	0
Untreated	650	509	228	0.5	2
Glycerol	650	509	228	0.5	2
Aluminate ester	650	509	228	0.5	2
Silane	650	509	228	0.5	2

**Table 4:** Tabulated shrinkage strain of different mixtures.

Mixture	Drying Shrinkage Strain (×10^−6^)											
	1 d	3 d	5 d	7 d	14 d	21 d	28 d	35 d	45 d	60 d	75 d	90 d
Control	25	59	83	95	152	183	205	229	241	263	274	279
Glycerol	20	39	56	71	121	154	173	189	196	208	219	223
Aluminates ester	23	42	65	81	140	167	189	211	224	241	251	256
Silane	26	64	89	107	168	198	219	246	268	279	296	304

**Table 5:** Initial water absorption coefficients of each mixture g/(m^2^h^1/2^).

Mixture	Sample 1	Sample 2	Sample 3	Average
Control	1426.9	1454.1	1271.0	1384.0
Glycerol	1584.7	1231.0	1163.9	1326.5
Aluminate ester	1091.8	1353.7	1334.6	1260.0
Saline	1464.4	1721.3	1737.4	1641.0

**Table 6:** Initial water absorption coefficients of each mixtures g/(m^2^h^1/2^).

Depth (mm)	Control	Glycerol	Aluminate Ester	Silane
1.5	0.284	0.354	0.273	0.32
3.0	0.362	0.456	0.344	0.41
4.5	0.293	0.334	0.281	0.31
6.0	0.192	0.225	0.185	0.21
7.5	0.120	0.146	0.117	0.13
9.0	0.105	0.097	0.079	0.085
11.5	0.074	0.072	0.061	0.074
14.0	0.054	0.061	0.052	0.061
16.5	0.051	0.052	0.045	0.054
19.0	0.041	0.042	0.034	0.044

**Table 7:** Mass loss and compressive strength loss after 25 freeze–thaw cycles.

Mixture	Initial Mass (g)	Final Mass (g)	Mass Loss (%)	Initial Compressive Strength (MPa)	Final Compressive Strength (MPa)	Strength Loss (%)
Control	2196.2	2189.2	0.32	52.3	49.6	5.2
Glycerol	2015.1	2012.7	0.12	46.4	39.8	14.2
Aluminate ester	1912.4	1909.9	0.13	35.6	34.2	1.4
Silane	1981.4	1974.1	0.37	39.7	34.9	12.1
